# Interventions and Their Effectiveness to Reduce HIV‐Related Stigma Among Women Living With HIV: A Systematic Review Protocol

**DOI:** 10.1002/hsr2.70671

**Published:** 2025-04-18

**Authors:** Zhongfang Yang, Yue Zhang, Lin Zhang, Shuyu Han, Zhijing Xu, Yan Hu

**Affiliations:** ^1^ School of Nursing, Fudan University Shanghai China; ^2^ JBI Fudan University Centre for Evidence‐based Nursing Shanghai China; ^3^ Shanghai Institute of Infectious Disease and Biosecurity Fudan University Shanghai China; ^4^ NYU Rory Meyers College of Nursing, New York University New York City USA; ^5^ Department of Nursing Shanghai Public Health Clinical Center Shanghai China; ^6^ School of Nursing, Peking University Beijing China

**Keywords:** gender equality, HIV/AIDS, reduced inequalities, stigma, well‐being, women's health

## Abstract

**Background:**

HIV‐related stigma is a significant barrier to accessing public health resources and achieving health equity in women living with HIV (WLWH). The complexity of eliminating stigma against WLWH is compounded by the dual characteristics of HIV and its chronic nature. In addition, WLWH often face weak social support. They experience social alienation and isolation. They also have difficulties in self‐identification and finding a sense of belonging within the new identity associated with their condition. These factors present considerable challenges to interventions aimed at combating HIV‐related stigma among WLWH. The purpose of this review is to identify interventions aimed at alleviating HIV‐related stigma among WLWH and evaluate their effectiveness.

**Methods:**

A systematic search of published studies from the inception of the databases to December 2024 will be conducted in China National Knowledge Infrastructure (CNKI), Wanfang Data, Chinese Scientific Journal Database (VIP), China Biomedical Literature Service System (SinoMed), MEDLINE (OVID), Embase(OVID), CINAHL (EBSCO), ProQuest, Scopus, Cochrane Library to evaluate the effects of health promotion interventions on HIV‐related stigma among WLWH. Two reviewers will independently assess the inclusion of each study, with any disagreements resolved by a third reviewer. Data will be extracted using predefined tables in accordance with best practices. Study quality will be assessed using the Effective Public Health Practice Project Quality Assessment Tool. A narrative synthesis of all studies will be presented, with appropriate statistical techniques applied to synthesize effectiveness data where feasible.

**Results:**

The results of this review will be disseminated through publication in a peer‐reviewed journal and shared online and on social media using accessible language and relevant infographics.

**Conclusion:**

This review evaluates health promotion interventions for reducing HIV‐related stigma among WLWH, identifying effective types and contexts. It addresses methodological strengths and weaknesses, providing policymakers insights into impactful interventions for global health and social care systems.

**Systematic Reviews Registration:**

The protocol was registered in PROSPERO (CRD42024571145).

## 1 Introduction

HIV disproportionately affects women worldwide compared to men, driven by both physiological and social factors [1]. Physiologically, women are more susceptible to HIV infection due to a larger mucosal surface exposed during sexual intercourse [1]. Social factors further exacerbate this vulnerability, including sexual violence, economic disadvantages, challenges in disclosing HIV status, limited control over sexual and reproductive decisions, and limited awareness of HIV infection [2]. In 2023, an estimated 39.9 million people [range: 36.1–44.6 million] were living with HIV globally, with women and girls comprising 53% of this population [3]. That year, 1.3 million new infections [range: 1–1.7 million] were reported, 44% of which occurred in women and girls.

Stigma refers to the social process through which certain individuals or groups are devalued or marginalized based on perceived differences or negative attributes [4]. HIV‐related stigma refers to the negative attitudes, beliefs, and discriminatory actions directed toward people living with HIV (PLWH) [5]. HIV‐related stigma is categorized into four types: enacted stigma (overt discrimination), anticipated stigma (fear of discrimination), perceived stigma (how PLWHA believe they are viewed), and internalized stigma (adopting negative societal attitudes) [6, 7, 8]. HIV‐related stigma manifests at two levels [9, 10]. Structural stigma includes systemic policies, practices, state and national laws, and regulations that marginalize PLWH, such as limited access to healthcare or employment discrimination. Community stigma involves negative attitudes and exclusionary behaviors from peers and local institutions. The mechanisms of HIV‐related stigma operate through the reinforcement of stereotypes, societal fear of contagion, and moral judgments that equate HIV with personal failure or immorality [11]. Intersectionality in HIV‐related stigma highlights how overlapping identities, such as gender, HIV status, and socioeconomic status, interact to exacerbate discrimination. For women living with HIV (WLWH)—referring to women diagnosed with HIV—intersecting factors amplify the stigma they face, as gender and societal norms often attribute greater blame to women for their HIV status, further marginalizing them within their communities [6, 7, 8]. Gender‐based stigma often reinforces patriarchal notions of morality, casting women as more culpable for their HIV status and leading to heightened social ostracization. Women may be labeled as promiscuous or immoral, a stigma that further intensifies the psychological burden of living with HIV, often greater than that faced by men [12]. HIV‐related stigma disproportionately hinders their ability to access timely HIV testing, adhere to antiretroviral therapy (ART), and achieve optimal health outcomes [13].

For WLWH, the challenges of reducing stigma are compounded by social and moral judgments associated with HIV and its chronic nature, requiring lifelong management [14]. These challenges are further exacerbated by psychosocial factors faced by WLWH, such as weak social support, social alienation, and isolation [15]. Moreover, traditional social and familial roles often hinder these women's self‐identification and sense of belonging within their HIV/AIDS identity [16]. In light of these challenges, interventions aimed at reducing HIV‐related stigma are essential [17].

These interventions can be targeted at multiple levels, as outlined by the socio‐ecological framework [18]. These levels include: (1) Intrapersonal level, where interventions target individuals to address internalized stigma through education and counseling [19, 20, 21, 22]. For example, cognitive‐behavioral therapy and self‐empowerment workshops have been used to help individuals cope with stigma; (2) Interpersonal level, with strategies aimed at enhancing social support and relationships to reduce stigma within families and social circles [22, 23, 24, 25, 26]. Examples include family counseling sessions and peer support networks; (3) Community level, with interventions promoting stigma reduction through public education campaigns and community engagement [10]. For instance, mass media campaigns and community theater performances have been used to educate communities about HIV and challenge stigmatizing attitudes; (4) Institutional level, with efforts to reform healthcare policies and practices to create stigma‐free environments [9]. Examples include healthcare worker training programs and stigma‐free certification initiatives for clinics; (5) Structural level, with advocacy for legislative changes and anti‐discrimination laws to address systemic stigma [27]. Examples include the enactment of anti‐discrimination policies and reforms in workplace practices to protect individuals living with HIV.

Previous systematic reviews showed that interventions generally had a positive effect on reducing HIV‐related stigma [28, 29, 30, 31, 32, 33, 34, 35, 36]. However, another systematic review concluded that the effects of these interventions were very limited [37], indicating the need for further systematic review to verify the robustness of the conclusions. Additionally, these reviews primarily focused on PLWH and their families [28, 31, 32, 33, 34, 35, 36], men who have sex with men (MSM) with and without HIV [30, 36], HIV‐uninfected individuals [35, 37], and medical institutions [29, 34, 35, 36], with no studies specifically examining interventions targeting stigma against WLWH [28, 29, 30, 31, 32, 33, 34, 35, 36, 37]. Furthermore, these reviews identified significant methodological limitations in the current research body, including small sample sizes, lack of control groups, inconsistent measurement of stigma outcomes, and limited long‐term follow‐up [28, 29, 30, 31, 32, 33, 34, 35, 36, 37].

Our team conducted a scoping review to systematically map existing interventions addressing HIV‐related stigma among WLWH [6, 7, 8]. The findings revealed a combination of effective and ineffective interventions, consistent with the conclusions of other systematic reviews. Additionally, our scoping review identified some gaps, particularly a lack of synthesis on the effectiveness of these interventions, underscoring the need for a comprehensive systematic review to evaluate their impact [6, 7, 8]. Addressing these knowledge gaps, the purpose of this systematic review is to identify interventions aimed at alleviating HIV‐related stigma among WLWH and evaluate their effectiveness.

## 2 Methods

The search strategy for this systematic review will be adapted from the scoping review conducted by Yang et al. [6, 7, 8]. This scoping review provides a comprehensive overview of existing interventions, which informs the development of inclusion criteria, the selection of key databases, and the identification of relevant search terms. Furthermore, outcome measures identified in the scoping review, such as changes in HIV‐related stigma levels, will be used to guide the selection of outcomes in this protocol.

### 2.1 Protocol and Registration

We will adhere to the Preferred Reporting Items for Systematic Reviews and Meta‐Analysis Protocols (PRISMA‐P) 2015 statement [38]. The complete PRISMA‐P checklist is available in Supporting Information S1: File 1. This protocol was registered with the PROSPERO International Prospective Register of Systematic Reviews (registration number CRD42024571145). The final review will be conducted in compliance with the PRISMA statement. Any major revisions to this protocol will be documented and published alongside the review findings.

### 2.2 Study Selection Criteria

#### 2.2.1 Type of Participants

Studies will be included if the participants are women diagnosed as HIV‐positive, encompassing both women living with HIV and women living with AIDS. Special populations such as drug addicts, individuals undergoing methadone maintenance treatment, alcoholics, and those with mental illnesses will be excluded from the study.

#### 2.2.2 Type of Intervention and Control Measures

Studies will only be included if the health promotion intervention is specifically to alleviate HIV‐related stigma among WLWH. The control group receive routine care or alternative interventions. Both the intervention and control groups receive identical care services in all other respects.

#### 2.2.3 Type of Outcome Measure

The outcome of interest is the incidence or severity of HIV‐related stigma measured using appropriate instruments. Both validated (e.g., 40‐item HIV Stigma Scale, 14‐item Stigma Scale for Chronic Illness, 28‐item Internalized HIV related Stigma Scale, 6‐item Internalized AIDS‐related Stigma Scale) and non‐validated outcome instruments of HIV‐related stigma will be considered. To be included, studies must report a quantitative measure of the effect of the health promotion intervention on HIV‐related stigma.

#### 2.2.4 Type of Studies

This systematic review will encompass studies published in a peer‐reviewed journal of doctoral thesis using a randomized controlled trials (RCTs) and quasi‐experimental studies (QES).

### 2.3 Search Strategy

#### 2.3.1 Electronic Databases

The selection of electronic databases and the development of the search strategy will be conducted in collaboration with information experts and will be informed by the search strategy used in a prior literature review [6, 7, 8]. The following electronic databases will be searched from the inception until December 2024: China National Knowledge Infrastructure (CNKI), Wanfang Data, Chinese Scientific Journal Database (VIP), China Biomedical Literature Service System (SinoMed), MEDLINE (OVID), Embase(OVID), CINAHL (EBSCO), ProQuest, Scopus, Cochrane Library. The literature included is available in Chinese or English. The search is not geographically restricted. The exact search terms used in all databases are detailed in Supporting Information S1: File 2.

#### 2.3.2 Manual Searches

We will examine the reference lists of studies included in this review and previous literature reviews on health promotion interventions aimed at reducing HIV‐related stigma to identify additional potentially relevant studies.

### 2.4 Study Selection

References will be managed using Zotero. One reviewer (Z.Y.) will remove duplicates. Subsequently, two reviewers (Z.Y. and Y.Z.) will independently assess each abstract to determine the necessity of a full‐text review. Any discrepancies between the two reviewers will be resolved by the third reviewer (Y.H.). The full text of potentially eligible studies will be retrieved, reviewed, and evaluated by two reviewers (Z.Y. and Y.Z.). A third reviewer (Y.H.) will be consulted if necessary. In accordance with PRISMA guidelines, a flowchart will be created to illustrate the selection process.

### 2.5 Data Extraction

Data extraction will be performed independently by two authors (Z.Y. and Y.Z.), and any disagreements will be resolved as previously described. The following information will be extracted using ‘the Cochrane Group Data collection form for intervention reviews’:

⇒ Study details: title, author, publication details, location, and language (if not English);
⇒ Study design: study type, duration, and outcomes measured;
⇒ Participant demographics: setting, inclusion and exclusion criteria, population size, and demographics;
⇒ Intervention characteristics: duration, type and mode of intervention;
⇒ Outcomes: incidence or severity of HIV‐related and assessment tools for evaluating the level of stigma or attitudes related to WLWH;
⇒ Results: raw data and effect sizes for HIV‐related stigma as the primary outcome and secondary outcomes;
⇒ Conclusions: conclusions of the authors and reviewers.

Detailed data extraction points can be found in Supporting Information S1: File 3.

### 2.6 Risk of Bias (Quality) Assessment

Two reviewers (Z.Y. and Y.Z.) will conduct an independent quality assessment of each study using the Effective Public Health Practice Project “Quality assessment tool for quantitative research” recommended by the Cochrane Public Health Group as it applies to experimental and quasi‐experimental study designs.

### 2.7 Description of Studies and Measurements of Effect Size

We anticipate encountering a variety of study designs and heterogeneous interventions targeting HIV‐related stigma among WLWH. Consequently, the data will focus on the effects of the interventions on HIV‐related stigma among WLWH.

We will further categorize studies by study design type (e.g., RCT, QES) and classify them according to intervention level (e.g., institutional level, individual level) and intervention type (e.g., information‐based intervention, technology‐based intervention, hybrid intervention). A narrative synthesis of all relevant studies will be provided by type outcome. This synthesis will be organized by study design and further broken down by intervention level and intervention type. It will describe study and participant characteristics, interventions, outcome measures, results, and authors’ conclusions.

The effectiveness of health promotion interventions in mitigating HIV‐related stigma in WLWH will be presented as mean effect sizes, such as standardized mean differences (SMDs), along with their corresponding confidence intervals (CIs). The rationale for using these summary statistics is the anticipated change in instruments assessing the same outcome. Effect sizes will be calculated using Hedges’ g, which provides a SMD along with 95% CI in studies with small sample sizes.

The main effect size for each study will be calculated from the first available post‐intervention measurement time point. In studies with multiple interventions, the main effect size will be calculated for the primary intervention group targeting HIV‐related stigma or for the group with the most robust design, i.e., the intervention that produces the greatest difference from the control group. For studies with multiple control groups, the main effect size will be calculated using the control group theoretically expected to produce the greatest difference from the intervention group. If there are more than two groups, we will initially perform pairwise comparisons and explore more complex analyses where appropriate, as recommended by Cochrane.

We will contact the authors of the studies included in this review to retrieve any missing data required for our analyses. We will attempt to calculate any missing SMDs for continuous measures from statistics reported in relevant papers, such as CIs and standard errors (SEs).

This study protocol includes the following pre‐specified analyses: (1) subgroup analyses by intervention type and level; (2) heterogeneity analysis using the *χ*² test (a priori significance level *p* < 0.1, 1‐sided) and *I*² statistic; and (3) sensitivity analyses to examine the robustness of the findings by excluding studies with high risk of bias. Any additional exploratory analyses will be conducted based on data trends observed during the analysis phase and will be clearly identified as exploratory. We will report the total number of studies by study design and intervention type using both fixed‐effects and random‐effects meta‐analyses. All statistical analyses will be conducted using R (version 4.4.1).

## 3 Conclusions

The 2024 UNAIDS theme highlights the critical importance of protecting the rights of women and girls to end AIDS. This systematic review focuses on evaluating the effectiveness of interventions aimed at reducing HIV‐related stigma among WLWH, a pressing issue given the profound impact of stigma on their mental health, social support, and adherence to treatment. Stigma exacerbates emotional distress, social isolation, and healthcare disparities, underscoring the need for targeted interventions to alleviate these negative effects and improve overall well‐being.

By identifying effective intervention types and the contexts in which they are most impactful, this review will provide valuable insights for policymakers. Policymakers play a crucial role in reducing stigma through supportive policies, resource allocation, and the promotion of evidence‐based interventions. Addressing stigma is not only a matter of social justice but also essential for achieving public health goals, such as increasing HIV treatment coverage and reducing transmission. With effective policy support, stigma‐reduction interventions can drive meaningful and sustainable improvements in both individual outcomes and broader public health effort.

## Author Contributions

**Zhongfang Yang:** conceptualization, methodology, software, data curation, formal analysis, funding acquisition, project administration, writing – original draft, writing – review and editing, visualization, resources. **Yue Zhang:** methodology, software, data curation. **Lin Zhang:** validation, supervision, writing – review and editing. **Shuyu Han:** validation, formal analysis. **Zhijing Xu:** software, validation. **Yan Hu:** conceptualization, methodology, writing – review and editing, funding acquisition, project administration, supervision, validation, resources.

## Ethics Statement

The authors have nothing to report.

## Conflicts of Interest

All authors have read and approved the final version of the manuscript. Yan Hu had full access to all of the data in this study and takes complete responsibility for the integrity of the data and the accuracy of the data analysis. The authors declare no conflicts of interest.

## Transparency Statement

The lead author Yan Hu affirms that this manuscript is an honest, accurate, and transparent account of the study being reported; that no important aspects of the study have been omitted; and that any discrepancies from the study as planned (and, if relevant, registered) have been explained.

## Supporting information

H supplementary file.

## Data Availability

This study is a systematic review protocol and does not involve the generation or analysis of primary data. Therefore, no data are available for sharing.
