# Supplementary material for: Interventions and Their Effectiveness to Reduce HIV‐Related Stigma Among Women Living With HIV: A Systematic Review Protocol
Source: Health Sci Rep. 2025 Apr 18;8(4):e70671. doi: 10.1002/hsr2.70671 (PMC12007456; doi:10.1002/hsr2.70671)
Supplement: Supplementary file 1 — H supplementary file. [file HSR2-8-e70671-s001.docx]

**Supplementary file 1: PRISMA-P 2015 checklist**

| **Section and topic** | **Item No.** | **Checklist Item** | **Reported on page#** |
| --- | --- | --- | --- |
| 1. **Administrative Information** | | | |
| Identification | 1a | Identify the report as a protocol of a systematic review | 1 |
| Update | 1b | Identify protocol as an update of a previous systematic review if applicable | n/a |
| Registration | 2 | Name of registry and registration number | 3+5 |
| 1. **Authors** | | | |
| Contact |  | Provide name, institutional affiliation, e-mail address of all protocol authors; provide physical mailing address of corresponding author | 1+2 |
| Contributions |  | Describe contributions of protocol authors and identify the guarantor of the review | 11 |
| Amendments |  | If the protocol represents an amendment of a previously completed or published protocol, identify as such and list changes; otherwise, state plan for documenting important protocol amendments | n/a |
| Support |  |  |  |
| - Sources | 5a | Indicate Sources of financial or other support for the review | 11 |
| - Sponsor | 5b | Provide name for the review funder and/or sponsor | 11 |
| - Role of sponsor of funder | 5c | Describe roles of funder(s), sponsor(s) and/or institution(s), if any, in developing the protocol | n/a |
| 1. **Introduction** | | | |
| Rationale | 6 | Describe the rationale for the review in the context of what is already known | 3-5 |
| Objectives | 7 | Provide an explicit statement of the question(s) the review will address with reference to participants, interventions, comparators, and outcomes (PICO) | 4+5 |
| 1. **Methods** | | | |
| Eligibility Criteria | 8 | Specify the study characteristics (such as PICO, study design, setting, time frame) and report characteristics (such as years considered, language, publication status) to be used as criteria for eligibility for the review | 5+6 |
| Information Sources | 9 | Describe all intended information sources (such as electronic databases, contact with study authors, trial registers or other grey literature sources) with planned dates of coverage | 6+7 |
| Search Strategy | 10 | Present draft of search strategy to be used for at least one electronic database, including planned limits, such that it could be repeated | 6+7+ Supplementary file 2 |
| 1. **Study Records** | | | |
| Data Management | 11a | Describe the mechanism(s) that will be used to manage records and data throughout the review | 7+8 |
| Selection Process | 11b | State the process that will be used for selecting studies (such as two independent reviewers) through each phase of the review (that is, screening, eligibility and inclusion in meta-analysis) | 7 |
| Data Collection Process | 11c | Describe planned method of extracting data from reports (such as piloting forms, done independently, in duplicate), any processes for obtaining and confirming data from investigators | 7+8+ Supplementary file 3 |
| Data Items | 12 | List and define all variables for which data will be sought (such as PICO items, funding sources), any pre-planned data assumptions and simplifications | 8-11 |
| Outcomes and prioritization | 13 | List and define all outcomes for which data will be sought, including prioritization of main and additional outcomes, with rationale | 8-10 |

**Supplementary file 2: Search strategy
Table A.2.1: MEDLINE (OVID)**

| **Searches** | **Search Terms** |
| --- | --- |
| 1 | HIV$.mp. or exp HIV/ |
| 2 | Immunodeficiency Virus, Human.mp. or exp HIV/ |
| 3 | Human Immunodeficiency Virus.mp. or exp HIV/ |
| 4 | Immunodeficiency Viruses, Human.mp. or exp HIV/ |
| 5 | Virus, Human Immunodeficiency.mp. or exp HIV/ |
| 6 | Viruses, Human Immunodeficiency.mp. or exp HIV/ |
| 7 | Human Immunodeficiency Viruses.mp. or exp HIV/ |
| 8 | Human T Cell Lymphotropic Virus Type III.mp. or exp HIV/ |
| 9 | Human T Cell Leukemia Virus Type III.mp. or exp HIV/ |
| 10 | LAV-HTLV-III.mp. or exp HIV/ |
| 11 | Lymphadenopathy Associated Virus.mp. or exp HIV/ |
| 12 | Lymphadenopathy Associated Viruses.mp. or exp HIV/ |
| 13 | Virus, Lymphadenopathy-Associated.mp. or exp HIV/ |
| 14 | Viruses, Lymphadenopathy-Associated.mp. or exp HIV/ |
| 15 | Human T Lymphotropic Virus Type III.mp. or exp HIV/ |
| 16 | AIDS Virus.mp. or exp HIV/ |
| 17 | AIDS Viruses.mp. or exp HIV/ |
| 18 | Virus, AIDS.mp. or exp HIV/ |
| 19 | Viruses, AIDS.mp. or exp HIV/ |
| 20 | Acquired Immune Deficiency Syndrome Virus.mp. or exp HIV/ |
| 21 | Acquired Immunodeficiency Syndrome Virus.mp. or exp HIV/ |
| 22 | HTLV-III.mp. or exp HIV/ |
| 23 | or/ 1- 22 |
| 24 | exp Women/ |
| 25 | Woman.mp. or exp Women/ |
| 26 | Women's Groups.mp. or exp Women/ |
| 27 | Women Groups.mp. or exp Women/ |
| 28 | Women's Group.mp. or exp Women/ |
| 29 | Or/ 24- 28 |
| 30 | 23 and 29 |
| 31 | exp Social Stigma/ |
| 32 | Social Stigmas.mp. or exp Social Stigma/ |
| 33 | Stigmas, Social.mp. or exp Social Stigma/ |
| 34 | Stigma, Social.mp. or exp Social Stigma/ |
| 35 | Or/ 31-34 |
| 36 | 30 and 35 |

**Table A.2.2: Embase (OVID)**

| **Searches** | **Search Terms** |  |
| --- | --- | --- |
| 1 | HIV.mp. or exp Human immunodeficiency virus/ |  |
| 2 | exp Human immunodeficiency virus infection/ or Immunodeficiency Virus, Human.mp. or exp Human immunodeficiency virus/ |  |
| 3 | Human Immunodeficiency Virus.mp. or exp Human immunodeficiency virus/ |  |
| 4 | exp Human immunodeficiency virus/ or Immunodeficiency Viruses, Human.mp. or exp Human immunodeficiency virus infection/ |  |
| 5 | exp Human immunodeficiency virus/ or exp Human immunodeficiency virus infection/ or Virus, Human Immunodeficiency.mp. |  |
| 6 | exp Human immunodeficiency virus infection/ or exp Human immunodeficiency virus/ or Viruses, Human Immunodeficiency.mp. |  |
| 7 | Human Immunodeficiency Viruses.mp. or exp Human immunodeficiency virus/ |  |
| 8 | Human T Cell Lymphotropic Virus Type III.mp. or exp Human immunodeficiency virus 1/ |  |
| 9 | Human T Cell Leukemia Virus Type III.mp. or exp Human immunodeficiency virus 1/ |  |
| 10 | exp Human immunodeficiency virus 1/ or exp acquired immune deficiency syndrome/ or LAV-HTLV-III.mp. or exp Human immunodeficiency virus infection/ |  |
| 11 | Lymphadenopathy Associated Virus.mp. or exp Human immunodeficiency virus/ |  |
| 12 | Lymphadenopathy Associated Viruses.mp. or exp Human immunodeficiency virus/ |  |
| 13 | Virus, Lymphadenopathy-Associated.mp. or exp Human immunodeficiency virus/ |  |
| 14 | Viruses, Lymphadenopathy-Associated.mp. or exp Human immunodeficiency virus/ |  |
| 15 | Human T Lymphotropic Virus Type III.mp. or exp Human immunodeficiency virus 1/ |  |
| 16 | AIDS Virus.mp. or exp Human immunodeficiency virus/ |  |
| 17 | AIDS Viruses.mp. or exp Human immunodeficiency virus/ |  |
| 18 | exp Human immunodeficiency virus infection/ or exp Human immunodeficiency virus/ or exp acquired immune deficiency syndrome/ or Virus, AIDS.mp. |  |
| 19 | exp acquired immune deficiency syndrome/ or exp Human immunodeficiency virus 1/ or Viruses, AIDS.mp. or exp Human immunodeficiency virus 1 infection/ |  |
| 20 | exp Human immunodeficiency virus infection/ or exp acquired immune deficiency syndrome/ or Acquired Immune Deficiency Syndrome Virus.mp. or exp Human immunodeficiency virus/ |  |
| 21 | exp Human immunodeficiency virus infection/ or exp acquired immune deficiency syndrome/ or exp Human immunodeficiency virus/ or Acquired Immunodeficiency Syndrome Virus.mp. or exp Human immunodeficiency virus 1/ |  |
| 22 | HTLV-III.mp. or exp Human immunodeficiency virus 1/ |  |
| 23 | Or/ 1-22 |  |
| 24 | Women.mp. or exp female/ |  |
| 25 | woman.mp. or exp female/ |  |
| 26 | exp female/ or Women's Groups.mp. or exp pregnancy/ |  |
| 27 | exp pregnancy/ or exp female/ or Women Groups.mp. |  |
| 28 | exp female/ or Women's Group.mp. or exp pregnancy/ |  |
| 29 | Or/ 24-28 |  |
| 30 | 23 and 29 |  |
| 31 | Social Stigma.mp. or exp social stigma/ |  |
| 32 | Social Stigmas.mp. or exp social stigma/ |  |
| 33 | exp stigma/ or Stigmas, Social.mp. or exp social stigma/ |  |
| 34 | exp social stigma/ or exp stigma/ or Stigma, Social.mp. |  |
| 35 | Or/ 31-34 |  |
| 36 | 30 and 35 |  |

**Table A.2.3: CINAHL (EBSCO)**

| **Searches** | **Search Terms** |
| --- | --- |
| S1 | (MM "Human Immunodeficiency Virus+") |
| S2 | "Immunodeficiency Virus, Human." |
| S3 | (MM "Human Immunodeficiency Virus+") OR "Human Immunodeficiency Virus" |
| S4 | "Immunodeficiency Viruses, Human" |
| S5 | "Virus, Human Immunodeficiency" |
| S6 | "Viruses, Human Immunodeficiency" |
| S7 | "Human Immunodeficiency Viruses" |
| S8 | "Human T Cell Lymphotropic Virus Type III" |
| S9 | "Human T-Cell Lymphotropic Virus Type III" |
| S10 | "Human T-Cell Leukemia Virus Type III" |
| S11 | "Human T Cell Leukemia Virus Type III" |
| S12 | "LAV-HTLV-III" |
| S13 | "Lymphadenopathy-Associated Virus" |
| S14 | "Lymphadenopathy Associated Virus" |
| S15 | "Lymphadenopathy-Associated Viruses" |
| S16 | "Virus, Lymphadenopathy-Associated" |
| S17 | "Viruses, Lymphadenopathy-Associated" |
| S18 | "Human T Lymphotropic Virus Type III" |
| S19 | "AIDS Virus" |
| S20 | "AIDS Viruses" |
| S21 | "Virus, AIDS" |
| S22 | "Viruses, AIDS" |
| S23 | (MM "Acquired Immunodeficiency Syndrome") OR "Acquired Immune Deficiency Syndrome Virus" |
| S24 | "Acquired Immunodeficiency Syndrome Virus" |
| S25 | "HTLV-III" |
| S26 | OR S1-S25 |
| S27 | (MM "Women+") OR "women" |
| S28 | "woman" |
| S29 | "Women's Groups" |
| S30 | "Women Groups" |
| S31 | "Women's Group" |
| S32 | "girl" |
| S33 | "girls" |
| S34 | OR S27-S33 |
| S35 | S26 AND S34 |
| S36 | "Social Stigma" |
| S37 | (MM "Stigma") OR "stigma" |
| S38 | "Social Stigmas" |
| S39 | "Stigmas, Social" |
| S40 | "Stigma, Social" |
| S41 | OR/S36-S40 |
| S42 | S35 AND S41 |
| Expanders - Also search within the full text of the articles; Apply equivalent subjects; Search modes - Boolean/Phrase  MM: To search for records containing the heading as a Major Subject | |

**Table A.2.4: ProQuest**

| **Set#** | **Search Terms** |
| --- | --- |
| S1 | HIV |
| S2 | Human Immunodeficiency Virus* |
| S3 | Human T?Cell Lymphotropic Virus Type III |
| S4 | Human T?Cell Leukemia Virus Type III |
| S5 | LAV-HTLV-III |
| S6 | Lymphadenopathy?Associated Virus* |
| S7 | Virus*, Lymphadenopathy-Associated |
| S8 | Human T Lymphotropic Virus Type III |
| S9 | AIDS Virus* |
| S10 | Acquired Immune Deficiency Syndrome Virus |
| S11 | Acquired Immunodeficiency Syndrome Virus |
| S12 | HTLV-III |
| S13 | OR [S1]-[S12] |
| S14 | Wom?n |
| S15 | Women Groups |
| S16 | Women's Group* |
| S17 | OR [S14] -[S16] |
| S18 | [S13] AND [S17] |
| S19 | Social Stigma* |
| S20 | [S18] AND [S19] |
| Databases: Health & Medical Collection, ProQuest Dissertations & Theses Global, ProQuest Dissertations & Theses Global A&I: The Humanities and Social Sciences Collection, ProQuest Dissertations & Theses Global A&I: The Sciences and Engineering Collection | |

**Table A.2.5: Scopus**

| **Search Terms** |
| --- |
| TITLE-ABS-KEY ( "HIV" ) OR TITLE-ABS-KEY ( "human immunodeficiency virus*" ) AND TITLE-ABS-KEY ( "stigma*" ) AND TITLE-ABS-KEY ( "wom?n" ) ) |

**Table A.2.6: The Cochrane Library**

| **Searches** | **Search Terms** |
| --- | --- |
| #1 | MeSH descriptor: [HIV] explode all trees |
| #2 | (Human Immunodeficiency Virus*):ti,ab,kw OR (Human T?Cell Leukemia Virus Type III):ti,ab,kw OR (Human T?Lymphotropic Virus Type III):ti,ab,kw OR (AIDS virus*):ti,ab,kw OR (Acquired Immune Deficiency Syndrome Virus*):ti,ab,kw (Word variations have been searched) |
| #3 | #1 OR #2 |
| #4 | MeSH descriptor: [Women] explode all trees |
| #5 | (women):ti,ab,kw OR (woman):ti,ab,kw OR (Women's Groups):ti,ab,kw OR (Women Groups):ti,ab,kw OR (Women's Group):ti,ab,kw (Word variations have been searched) |
| #6 | #4 OR #5 |
| #7 | #3 AND #6 |
| #8 | MeSH descriptor: [Social Stigma] explode all trees |
| #9 | (Social Stigma*):ti,ab,kw OR (Social Stigma*):ti,ab,kw OR (Stigma*, Social):ti,ab,kw OR (Stigma*, Social):ti,ab,kw (Word variations have been searched) |
| #10 | #8 OR #9 |
| #11 | #7 AND #10 |
|  | Limit by date: all dates |
| ti: title; ab: abstract; kw: keyword | |

**Supplementary file 3: Pro-form for Data Extraction**

| **Reviewer** |  |
| --- | --- |
| **Date form completed** |  |

**Study Details**

| **Title** |  |
| --- | --- |
| **Author** |  |
| **Year Published** |  |
| **Journal** |  |
| **Location (country/city)** |  |
| **Language (if not English)** |  |

|  |  | **Location in text**  **(page/figure/table/other)** |
| --- | --- | --- |
| **Type of study** | Randomised controlled trial ¨  Non-randomised controlled trial ¨  Controlled before and after study ¨  Uncontrolled before and after study ¨ |  |
| 1. **Start Date** 2. **EndDate** | A.  B. |  |
| **Outcomes Measured (aims)** |  |  |

**Participants**

|  | **Location in text (page/figure/table/other)** |
| --- | --- |
| **Setting** | Large population in unspecified setting ¨  Community: ............................................... ¨  Supported living ¨ Other: .......................................................... |
| **Type of Participants** |  |
| **Enrolment Eligibility**   1. **Inclusion Criteria** 2. **Exclusion Criteria** | A.  B. |
| **Recruitment process (e.g. phone, mail, clinic)** |  |
| **Total number randomised (or total population for non-RCT)** |  |
| **Age range (mean age)** |  |
| **Gender (% female)** |  |
| **Ethnicity** |  |
| **Sample size** |  |
| **Any other relevant sociodemographic information** |  |
| **Any subgroups established** |  |
| **Notes** |  |

**Intervention**

|  |  | | **Location in text (page/figure/table/other)** |
| --- | --- | --- | --- |
| **Intervention** | Description | |  |
|  | Duration of each intervention episode | |  |
|  | Duration of intervention studied (total study duration) | |  |
|  | Frequency (daily/weekly etc.) | |  |
|  | Provider (i.e. no., profession, training) | |  |
|  | Economic information (cost, resource requirements) | |  |
| **Type of intervention** | Information Training ¨  Skill Training ¨ | |  |
| **Level of Intervention** | Individual Level | Non-infected population ¨  Infected population ¨ |  |
|  | Institutional Level | Hospital ¨  Clinic ¨  Community ¨  other ........................................ ¨ |  |
| Compliance |  |  |  |
| Notes |  |  |  |

**Outcomes**

|  | **Description as stated in paper/report** | **Location in text (page/figure/table/other)** |
| --- | --- | --- |
| **Measure of HIV-related stigma** |  |  |
| **Outcome tool validated** | Yes ¨ No ¨ Unclear ¨ |  |
| **Any outcomes other than stigma** | Yes ¨ Details ..................  No ¨ |  |

**Results: effect of the intervention on HIV-related stigma**

|  |  | | | | | | **Location in text (page/figure/table/other)** |
| --- | --- | --- | --- | --- | --- | --- | --- |
| **Comparison** |  | | | | | |  |
| **Outcome** |  | | | | | |  |
| **Subgroup** |  | | | | | |  |
| **Time point (from start or end of intervention)** |  | | | | | |  |
| **Results** | Intervention | | | comparison | | |  |
|  | Mean | SD (or other variance, specify) | No participants | Mean | SD (or other variance, specify) | No participants |  |
|  |  |  |  |  |  |  |  |
| **Effect size** | Effect size: ___________________________________ Standard error: _______________________________ Inverse variance:______________________________ 95% confidence interval: ________________________ | | | | | |  |
| **Any other results reported (e.g. Odds ratio)** |  | | | | | |  |
| **Statistical method used** |  | | | | | |  |
| **Notes** |  | | | | | |  |

**Results: effects of the intervention on other outcome continuous variables**

|  |  | | | | | | **Location in text (page/figure/table/other)** |
| --- | --- | --- | --- | --- | --- | --- | --- |
| **Comparison** |  | | | | | |  |
| **Outcome** |  | | | | | |  |
| **Subgroup** |  | | | | | |  |
| **Time point (from start or end of intervention)** |  | | | | | |  |
| **Results** | Intervention | | | comparison | | |  |
|  | Mean | SD (or other variance, specify) | No participants | Mean | SD (or other variance, specify) | No participants |  |
|  |  |  |  |  |  |  |  |
| **Effect size** | Effect size: ___________________________________ Standard error: _______________________________ Inverse variance:______________________________ 95% confidence interval: ________________________ | | | | | |  |
| **Any other results reported (e.g. Odds ratio)** |  | | | | | |  |
| **Statistical method used** |  | | | | | |  |
| **Notes** |  | | | | | |  |
